# Impact of immune checkpoint gene *CD155* Ala67Thr and *CD226* Gly307Ser polymorphisms on small cell lung cancer clinical outcome

**DOI:** 10.1038/s41598-021-81260-1

**Published:** 2021-01-19

**Authors:** Jang Hyuck Lee, Seung Soo Yoo, Mi Jeong Hong, Jin Eun Choi, Soyoun Kim, Hyo-Gyoung Kang, Sook Kyung Do, Ji Hyun Kim, Sun Ah Baek, Won Kee Lee, Jae Do Yoo, Sun Ha Choi, Yong Hoon Lee, Hyewon Seo, Jaehee Lee, Shin Yup Lee, Seung Ick Cha, Chang Ho Kim, Jae Yong Park

**Affiliations:** 1 Department of Biochemistry and Cell Biology, School of Medicine, Kyungpook National University, Daegu, 41944 Republic of Korea; 2 BK21 Plus KNU Biomedical Convergence Program, Department of Biomedical Science, Kyungpook National University, Daegu, 41944 Republic of Korea; 3 Department of Internal Medicine, School of Medicine, Kyungpook National University Hospital, Kyungpook National University, 807, Hoguk-ro, Buk-gu, Daegu, 41404 Republic of Korea; 4 Cell and Matrix Research Institute, School of Medicine, Kyungpook National University, Daegu, 41944 Republic of Korea; 5 Tumor Heterogeneity and Network (THEN) Research Center, School of Medicine, Kyungpook National University, Daegu, 41944 Republic of Korea; 6 Collaboration Center, Kyungpook National University, Daegu, 41944 Republic of Korea

**Keywords:** Cancer genetics, Lung cancer, Genetic markers, Genotype

## Abstract

This study was conducted to investigate the impact of genetic variants of immune checkpoint genes on the treatment outcome in small cell lung cancer (SCLC). In the present study, 261 platinum doublet-treated SCLC patients were enrolled. A total of 96 polymorphisms in 33 immune checkpoint-related genes were selected, and their association with chemotherapy response and survival outcomes were analyzed. Among the polymorphisms studied, *CD155* rs1058402G > A (Ala67Thr, A67T) and *CD226* rs763361C > T (Gly307Ser, G307S) were significantly associated with SCLC treatment outcome. The rs1058402G > A had a worse chemotherapy response and overall survival (under a dominant model, adjusted odds ratio [aOR] = 0.52, 95% confidence interval [CI] = 0.27–0.99, *P* = 0.05; adjusted hazard ratio [aHR] = 1.55, 95% CI = 1.12–2.14, *P* = 0.01, respectively). The rs763361C > T had better chemotherapy response and overall survival (under a dominant model, aOR = 2.03, 95% CI = 1.10–3.75, *P* = 0.02; aHR = 0.69, 95% CI = 0.51–0.94, *P* = 0.02, respectively). When the rs1058402GA/AA and rs763361CC genotypes were combined, the chemotherapy response and overall survival were significantly decreased as the number of bad genotypes increased (aOR = 0.52, 95% CI = 0.33–0.81, *P*trend = 0.004; aHR = 1.48, 95% CI = 1.19–1.84, *P*trend = 4 × 10^−4^, respectively). The 3-D structural model showed that *CD155* A67T created a new hydrogen bond and structural change on *CD155*. These changes resulted in extending the distance and losing the hydrogen bonds between *CD155* and *CD226*, thus weakening *CD155*/*CD226* binding activity. In conclusion, *CD155* rs1058402G > A and *CD226* rs763361C > T may be useful for predicting the clinical outcomes of SCLC patients after chemotherapy.

## Introduction

Lung cancer is still the leading cause of cancer-related deaths^1^. Approximately 15% of lung cancer is categorized as small cell lung cancer (SCLC), which is characterized as having a more rapid doubling time, higher growth rate, and earlier metastasis than non-small cell lung cancer (NSCLC)^2^. Smoking is the most potent known cause of SCLC^2^. One-third of patients with SCLC are diagnosed with limited-stage disease if the cancer is confined to the ipsilateral hemithorax. The others are diagnosed with extensive-stage disease if it is beyond the ipsilateral hemithorax, including distant metastases. The development of SCLC therapy has been stagnant for decades, although there has been recent improvement in the treatment of NSCLC^3^. The combined modality treatment with chemotherapy and radiotherapy is the current treatment standard for limited-stage SCLC^2^. Chemotherapy with platinum doublet is the cornerstone of therapy for extensive-stage SCLC. SCLC is very sensitive to initial chemotherapy, but most SCLC patients relapse and eventually die. Therefore, efforts are needed to predict chemotherapy responses and find prognostic markers.

The immune system defends our bodies from infectious organisms and other invaders. The immune system is also important in preventing and eradicating cancers^4^. In general, immune cells can recognize tumor cells and destroy them. However, cancer cells have developed ways of escaping the immune system by suppressing the activation of immune cells^5^. Therefore, cancer cells can survive and spread beyond the patient’s immune system. Many researchers have tried to control immune-escaping tumors^5–7^. Immune checkpoint inhibitors are drugs that can block inhibitor signals from cancer cells, restore the immune system, and reactivate immune cells to kill cancer cells. Recently, immune checkpoint inhibitors have been actively used in the treatment of cancers, including lung cancer^8–11^.

The genetic variants in immune checkpoints may affect the body’s response to chemotherapy or lung cancer prognosis, considering the immune system’s ability to prevent and kill cancer cells. We previously reported that genetic variants in immune checkpoint genes were associated with the prognosis of surgically resected NSCLC^12^. Programmed cell death-ligand 1 (PD-L1) polymorphisms were associated with chemotherapy response and clinical outcomes of advanced NSCLC^13^. Nomizo et al. reported that the polymorphisms in immune checkpoint genes had an effect on the response rate and progression survival in NSCLC patients with nivolumab treatment^14^. However, only a few studies had been conducted regarding SCLC. Therefore, we investigated the effects of genetic variants in immune checkpoint genes on the chemotherapy response and prognosis in SCLC.

## Materials and methods

### Study population

**Figure 1:**
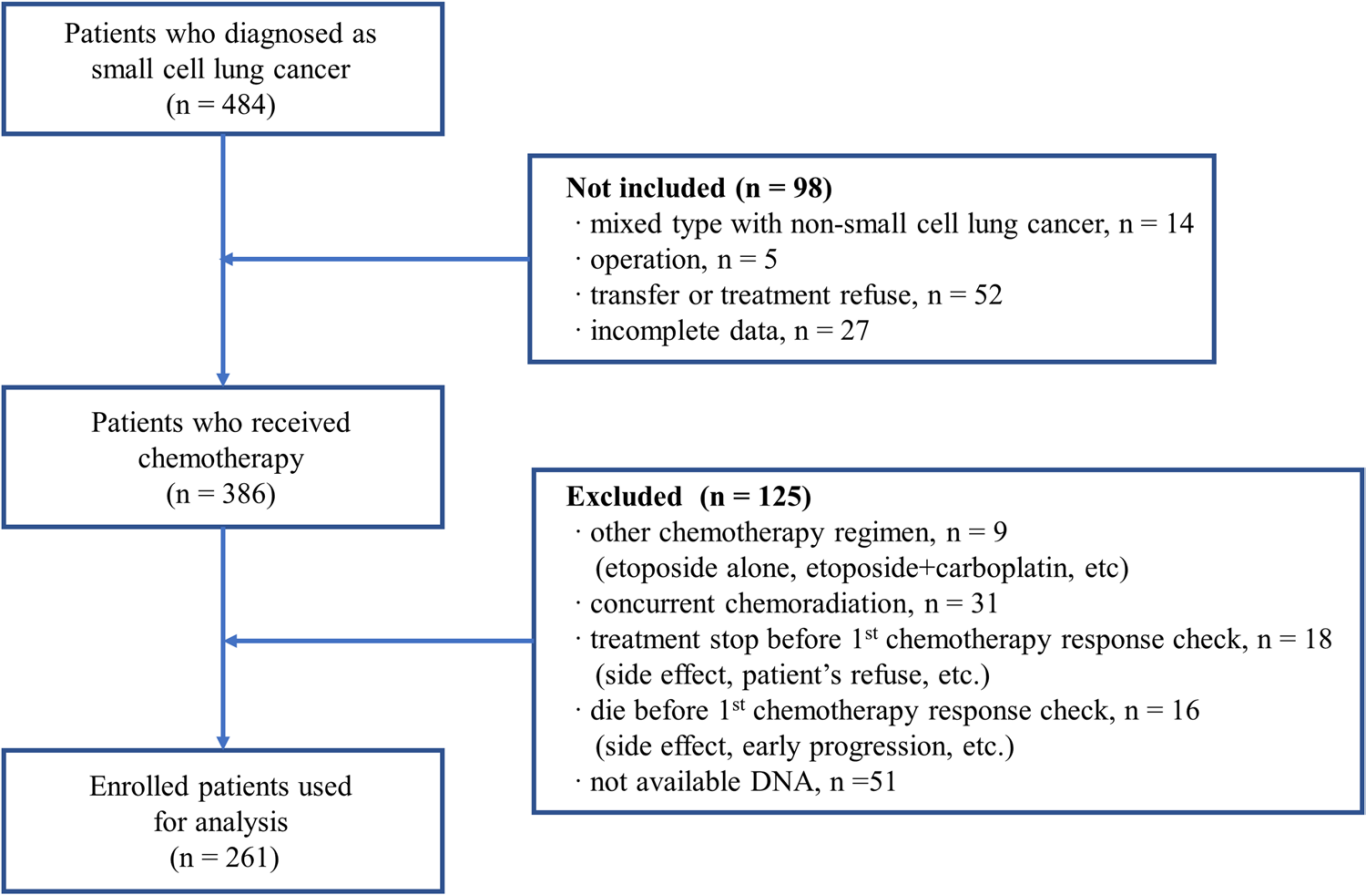
Flow diagram of patients selection.

This study is observational retrospective study. This study included 261 patients diagnosed with SCLC at Kyungpook National University Hospital (KNUH) in Daegu, Korea between March 2001 and November 2017. The flow of selection of the patients is shown in Fig. 1. All the patients were treated with at least two cycles of platinum doublet chemotherapy as first-line treatment. Patients with limited-stage SCLC who underwent chemotherapy with concurrent radiotherapy were excluded to avoid the confounding effect of radiation on the chemotherapy response. If radiotherapy was conducted sequentially after 2 cycles of chemotherapy, these patients were included. In this case, the response after 2 cycles of chemotherapy was considered as the best response to chemotherapy. There were two chemotherapy regimens. One consists of cisplatin 60 mg/m^2^ on day 1 and etoposide 100 mg/m^2^ on day 1, 2, and 3 every 3 weeks. The other consists of cisplatin 60 mg/m^2^ on day 1 and irinotecan 60 mg/m^2^ on day 1, 8, and 15 every 4 weeks. The treatment was discontinued in case of disease progression, major toxicities, or according to patient’s or physician’s decision. Response assessment was carried out every two cycles of chemotherapy using the Response Evaluation Criteria in Solid Tumors^15^. Chemotherapy response fell into two categories: responder and non-responder. Responder means the best response was a complete response or a partial response. A stable disease or progressive disease was defined as non-responder.

This study was approved by the Institutional Review Boards of KNUH. The blood samples for genotyping were provided by the National Biobank of KNUH, which is supported by the Ministry of Health, Welfare, and Family Affairs. All blood samples were obtained before the 1st chemotherapy session. All subjects are 18 years of age or older and informed consent was obtained prior to chemotherapy.

### Polymorphism selection and genotyping

We selected 38 genes involved in the immune checkpoint pathway by searching public databases and related literatures^16–18^. To collect polymorphisms in immune checkpoint genes, we searched the public single-nucleotide polymorphisms (SNPs) database (http://www.ncbi.nlm.nih.gov/SNP) and RegulomeDB (http://www.regulomedb.org/). After excluding minor allele frequencies of ≤ 0.05 by the HapMap JPT data, 216 potentially functional SNPs were collected using the FuncPred utility for functional SNP prediction in the SNPinfo web server (https://snpinfo.niehs.nih.gov/). After excluding those in linkage disequilibrium (LD) (r^2^ ≥ 0.8) using the TagSNP utility for LD tag SNP selection, 123 SNPs were selected for genotyping. Among the 123 SNPs, 27 of them with call rates of < 95% or *P* value for Hardy–Weinberg equilibrium (HWE) of < 0.05 were excluded from further analysis. Finally, the remaining 96 SNPs in 33 immune checkpoint genes were analyzed for the association and response study (Supplementary Table S1). Genotyping was performed using Sequenom MassARRAY iPLEX Platform (Agena Bioscience, San Diego, USA). All genotyping was conducted blindly with respect to patient status. All methods were performed in accordance with the relevant guidelines and regulations.

### Statistical analysis

The HWE was tested using a goodness-of-fit χ^2^ test with one degree of freedom. For comparison between clinical variables or genotypes and chemotherapy response, the odds ratio (OR) and 95% confidence interval (CI) were calculated using unconditional logistic regression analysis. Overall survival (OS) was counted from the 1st chemotherapy session date to death or last follow-up. Progression-free survival (PFS) was measured from the day of 1st chemotherapy until disease progression or death from any cause. Kaplan–Meier method was used to estimate the survival outcome. Log-rank test was used to compare OS across different groups or genotypes. Multivariate Cox proportional hazards models were used to estimate the hazard ratio (HR) and 95% CIs. Adjusting factors were age, gender, smoking status, stage, Eastern Cooperative Oncology Group (ECOG) performance status, weight loss, neuron specific enolase (NSE) level, 1st chemotherapy regimen, second-line chemotherapy, and radiation to primary tumor. A *P* value of less than 0.05 was considered statistically significant. Statistical analyses were performed using the Statistical Analysis System for Windows, version 9.4 (SAS Institute, Cary, NC, USA).

### Structure modeling of CD155 A67T variant.

In order to evaluate the effect of the rs1058402G > A of CD155, which substitutes alanine with threonine at codon 67, The mutant structure of CD155(A67T) was modeled using MODELLER v9.12 with the crystal structures of wildtype CD155 (PDB : 6ISC). The least violated 10 structures were further refined by model/refine loops (loop modeling protocol DOPE) using UCSF Chimera v1.14 and a structure showed no violation and the lowest energy was selected as a model. All images of CD226/CD155 (wild type *vs.* A67T mutant) were made in PyMOL (https://pymol.org/2/).

## Results

### Patient characteristics and clinical predictors

Chemotherapy response and OS according to patients’ clinical characteristics are shown in Table 1. The overall response rate was 72.8% (95% CI = 67.4–78.2%). The median survival time (MST) was 10.4 months (95% CI = 9.1–11.1 months). The response rate was higher in the irinotecan/cisplatin regimen than the etoposide/cisplatin regimen (78.7% vs. 67.2%, *P* = 0.04), but OS was not different based on regimens (MST, 10.0 months vs. 10.7 months, *P* = 0.88, Table 1). OS was associated with age, stage, ECOG performance status, weight loss, NSE level, second-line chemotherapy, and radiation to tumor (Table 1).

**Table 1:** Univariate analysis for chemotherapy response and overall survival by clinical variables.

Variables	No. of case	Chemotherapy response				Overall survival				
		Responder	Non-responder	OR (95% CI)	*P*	MST	95% CI	Log-Rank *P*	HR (95% CI)	*P*
		(CR + PR)^a^	(SD + PD)^a^			(month)				
Overall	261	190 (72.8)^b^	71 (27.2)^b^			10.4	9.1–11.1			
**Age (year)**										
< 68	129	100 (77.5)	29 (22.5)	1		11.6	10.7–13.0		1	
≥ 68	132	90 (68.2)	42 (31.8)	0.62 (0.36–1.08)	0.09	7.8	6.6–8.8	1 × 10^–4^	1.60 (1.25–2.05)	2 × 10^–4^
**Gender**										
Male	226	163 (72.1)	63 (27.9)	1		10.3	9.0–11.2		1	
Female	35	27 (77.1)	8 (22.9)	1.30 (0.56–3.02)	0.54	10.8	6.3–15.0	0.75	0.94 (063–1.40)	0.75
**Smoking status**										
Never	19	16 (84.2)	3 (15.8)	1		11.2	6.3–15.2		1	
Ever	242	174 (71.9)	68 (28.1)	0.48 (0.14–1.70)	0.26	10.1	9.0–11.1	0.82	1.03 (0.64–1.76)	0.82
**Stage**										
LD	66	46 (69.7)	20 (30.3)	1		12.8	10.6–15.2		1	
ED	195	144 (73.8)	51 (26.2)	1.23 (0.66–2.27)	0.51	9.4	8.1–10.7	0.001	1.67 (1.21–2.30)	0.002
**ECOG**										
0–1	216	162 (75.3)	53 (24.7)	1		10.4	9.1–11.3		1	
2	45	28 (60.9)	18 (39.1)	0.51 (0.26–0.99)	0.05	7.1	4.3–9.0	3 × 10^–4^	1.81 (1.31–2.53)	4 × 10^–4^
**Weight loss**^**c**^										
No	184	139 (75.1)	46 (24.9)	1		11.1	10.0–11.9		1	
Yes	77	51 (67.1)	25 (32.9)	0.68 (0.38–1.21)	0.19	8.0	7.0–10.0	0.01	1.42 (1.07–1.89)	0.02
**NSE**										
< 14.7	96	66 (68.8)	30 (31.2)	1		11.2	10.0–13.7		1	
≥ 14.7	147	109 (74.2)	38 (25.8)	1.30 (0.74–2.30)	0.36	9.2	7.5–10.3	0.02	1.41 (1.07–1.87)	0.02
**Regimen**										
EP	134	90 (67.2)	44 (32.8)			10.7	8.8–12.2			
IP	127	100 (78.7)	27 (21.3)	1.81 (1.04–3.16)	0.04	10.0	8.7–11.2	0.88	0.98 (0.75–1.28)	0.88
**Second line chemotherapy**										
No	121					7.1	6.1–8.2		1	
Yes	140					11.9	11.0–13.5	1 × 10^–5^	0.56 (0.43–0.73)	2 × 10^–5^
**Radiation to tumor**										
No	227					9.5	8.1–10.6		1	
Yes	34					16.4	12.8–null	2 × 10^–5^	0.33 (0.20–0.56)	4 × 10^–4^

CR, complete response; PR, partial response; SD, stable disease; PD, progressive disease; OR, odds ratio; CI, confidence interval; MST, median survival time; HR, hazard ratio; LD, Limited disease; ED, Extensive disease; ECOG, Eastern Cooperative Oncology Group; NSE, neuron specific enolase; EP, etoposide-cisplatin; IP, irinotecan-cisplatin.

^a^Number of patients: 18 CR, 172 PR, 49 SD, and 22 PD.

^b^Row percentage.

^c^Unintentional weight loss > 5% within 3 months.

### Effect of polymorphisms on treatment outcome

**Figure 2:**
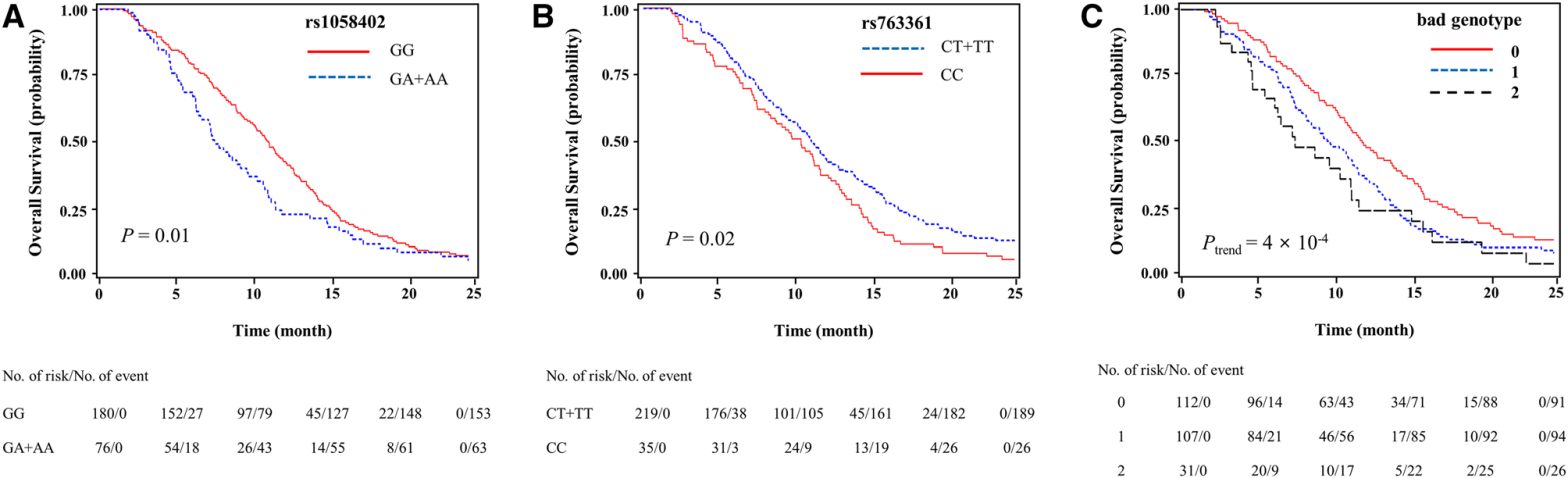
Kaplan–Meier plots for overall survival according to polymorphisms. (**A**) *CD155* rs1058402, (**B**) *CD226* rs763361, and (**C**) combined bad genotypes. *P* values were calculated using multivariate Cox proportional hazard models.

Among the observed 96 polymorphisms, *CD155* rs1058402G > A (Ala67Thr, A67T) and *CD226* rs763361C > T (Gly307Ser, G307S) were associated with both chemotherapy response and OS. The rs1058402G > A was significantly associated with worse chemotherapy response and OS (under a dominant model, adjusted OR [aOR] = 0.52, 95% CI = 0.27–0.99, *P* = 0.05 and adjusted HR [aHR] = 1.55, 95% CI = 1.12–2.14, *P* = 0.01; Table 2 and Fig. 2A). The effect of rs1058402 on PFS had the same trend as OS, although it was not statistically significant (under a dominant model, aHR = 1.30, 95% CI = 0.96–1.76, *P* = 0.09). The rs763361C > T showed significantly better chemotherapy response, OS, and PFS, respectively (under a dominant model, adjusted aOR = 2.03, 95% CI = 1.10–3.75, *P* = 0.02; aHR = 0.69, 95% CI = 0.51–0.94, *P* = 0.02; aHR = 0.73, 95% CI = 0.54–0.97, *P* = 0.03, respectively, Table 2 and Fig. 2B).

**Table 2:** Chemotherapy response and survival outcomes according to *CD155* rs1058402 and *CD226* rs763361 genotypes.

Polymorphism	No. of case (%)^a^	Chemotherapy response				Overall survival				Progression-free survival		
		Responder (%)^b^	Non-responder (%)^b^	OR (95% CI)^c^	*P*^c^	MST (95% CI)^d^	Log-Rank *P*	HR (95% CI)^d^	*P*^d^	Log-Rank *P*	HR (95% CI)^d^	*P*^d^
**rs1058402**												
GG	180 (70.3)	137 (76.1)	43 (23.9)	1.00		10.8 (9.9–12.3)		1.00			1	
GA	66 (25.8)	43 (65.2)	23 (34.8)	0.51 (0.26–1.00)	0.05	7.7 (6.3–10.1)		1.56 (1.11–2.18)	0.01		1.30 (0.95–1.79)	0.11
AA	10 ( 3.9)	7 (70.0)	3 (30.0)	0.57 (0.13–2.56)	0.47	7.9 (2.5–17.9)	0.12	1.52 (0.73–3.18)	0.27	0.39	1.29 (0.62–2.67)	0.5
Dominant				0.52 (0.27–0.99)	0.05	7.7 (6.3–10.1)	0.07	1.55 (1.12–2.14)	0.01	0.47	1.30 (0.96–1.76)	0.09
Recessive				0.71 (0.16–3.09)	0.64	10.4 (9.0–11.2)	0.80	1.33 (0.64–2.76)	0.45	0.42	1.20 (0.58–2.47)	0.63
Codominant				0.61 (0.36–1.04)	0.07			1.39 (1.07–1.80)	0.01		1.22 (0.95–1.57)	0.11
**rs763361**												
CC	97 (38.2)	66 (68.0)	31 (32.0)	1.00		10.0 (8.0–11.2)		1.00			1	
CT	122 (48.0)	93 (76.2)	29 (23.8)	1.80 (0.94–3.45)	0.08	10.0 (8.7–11.2)		0.72 (0.52–0.99)	0.04		0.74 (0.55–1.01)	0.06
TT	35 (13.8)	29 (82.9)	6 (17.1)	3.10 (1.10–8.75)	0.03	13.4 (10.5–15.4)	0.04	0.61 (0.38–0.98)	0.04	0.33	0.66 (0.43–1.03)	0.07
Dominant				2.03 (1.10–3.75)	0.02	10.7 (9.2–11.7)	0.04	0.69 (0.51–0.94)	0.02	0.28	0.73 (0.54–0.97)	0.03
Recessive				2.27 (0.85–6.09)	0.10	10.0 (8.8–10.8)	0.05	0.75 (0.48–1.15)	0.19	0.19	0.80 (0.54–1.19)	0.27
Codominant				1.78 (1.12–2.82)	0.02			0.76 (0.61–0.96)	0.02		0.80 (0.64–0.98)	0.03

OR, odds ratio; CI, confidence interval; MST, median survival time (months); HR, hazard ratio.

^a^Column percentage.

^b^Row percentage.

^c^OR, 95% CI, and their corresponding *P*-values were calculated by multivariate regression analysis, adjusted for age, gender, smoking status, stage, Eastern Cooperative Oncology Group performance status, weight loss, neuron specific enolase level, and first chemotherapy regimen.

^d^HR, 95% CI and their corresponding *P*-values were calculated using multivariate Cox proportional hazard models, adjusted for age, gender, smoking status, stage, Eastern Cooperative Oncology Group performance status, weight loss, neuron specific enolase level, first chemotherapy regimen, second line chemotherapy, and radiation to primary tumor.

We also analyzed survival outcomes in the extensive-stage only. In the extensive-stage SCLC, both rs1058402 and rs763361 were associated with OS in univariated analysis (under a dominant model, Log Rank *P* = 0.03 and 0.006, respectively), but only rs1058402 was associated with OS in multivariated analysis (under a dominant model, aHR = 1.75, 95% CI = 1.21–2.53, *P* = 0.003) (Supplementary Table S2). PFS showed the same trend as OS, although it was not statistically significant (under a dominant model, aHR for rs1058402 = 1.34, 95% CI = 0.94–1.90, *P* = 0.11; aHR for rs763361 = 0.79, 95% CI = 0.56–1.10, *P* = 0.16, respectively, Supplementary Table S2).

The effects of the two variants on chemotherapy response and OS did not differ according to clinical variables, such as age, gender, etc., when data were categorized by these factors, except for chemotherapy regimen on chemotherapy response (*P* value for homogeneity test > 0.05, Supplementary Table S3).

### Combined effects of rs1058402 and rs763361 on treatment outcomes

The rs1058402GA/AA and rs763361CC genotypes were associated with worse chemotherapy response and OS. When these genotypes were considered bad genotypes, chemotherapy response was decreased as the number of bad genotypes increased (responder 80.4% with 0 bad genotype, 71.0% with 1 bad genotype, and 61.3% with 2 bad genotypes; aOR = 0.52, 95% CI = 0.33–0.81, *P*trend = 0.004; Table 3). The MST was also significantly decreased as the number of bad genotypes increased (MST = 11.5 months with 0 bad genotype, 9.5 months with 1 bad genotype, and 7.2 months with 2 bad genotypes; aHR = 1.48, 95% CI = 1.19–1.84, *P*trend = 4 × 10^−4^; Table 3 and Fig. 2C). PFS also significantly decreased as the number of bad genotypes increased (*P*trend = 0.009). In an analysis of only patients with extensive-stage, the combined effect of rs1058402 and rs763361 was still significant in chemotherapy response, OS, and PFS, respectively (aOR = 0.55, 95% CI = 0.33–0.93, *P*trend = 0.03; aHR = 1.55, 95% CI = 1.21–1.98, *P*trend = 6 × 10^–4^: aHR = 1.28, 95% CI = 1.07–1.59, *P*trend = 0.04, respectively, Supplementary Table S4).

**Table 3:** Combined effects of rs1058402G > A and rs763361C > T genotypes on chemotherapy response and survival outcome.

No. of bad genotype^a^	No. of case (%)^b^	Chemotherapy response				Overall survival			Progression-free survival	
		Responder (%)^c^	Non-responder (%)^c^	OR (95% CI)^d^	*P*^d^	MST (95% CI)	HR (95% CI)^e^	*P*^e^	HR (95% CI)^e^	*P*^e^
0	112 (44.8)	90 (80.4)	22 (19.6)	1.00		11.5 (10.1–13.4)	1.00		1.00	
1	107 (42.8)	76 (71.0)	31 (29.0)	0.55 (0.29–1.04)	0.01	9.5 (7.7–11.0)	1.67 (1.24–2.25)	0.001	1.57 (1.16–2.12)	0.004
2	31 (12.4)	19 (61.3)	12 (38.7)	0.31 (0.13–0.78)	0.01	7.2 (4.6–10.7)	1.99 (1.25–3.17)	0.004	1.50 (0.96–2.34)	0.08
*P*_trend_				0.52 (0.33–0.81)	0.004		1.48 (1.19–1.84)	4 × 10^–4^	1.30 (1.07–1.59)	0.009

OR, odds ratio; CI, confidence interval; MST, median survival time (months); HR, hazard ratio.

^a^Bad genotype; rs1058402GA/AA and rs763361CC.

^b^Column percentage.

^c^Row percentage.

^d^OR, 95% CI, and their corresponding *P*-values were calculated by multivariate regression analysis, adjusted for age, gender, smoking status, stage, Eastern Cooperative Oncology Group performance status, weight loss, neuron specific enolase level, and first chemotherapy regimen.

^e^HR, 95% CI and their corresponding *P*-values were calculated using multivariate Cox proportional hazard models, adjusted for age, gender, smoking status, stage, Eastern Cooperative Oncology Group performance status, weight loss, neuron specific enolase level, first chemotherapy regimen, second line chemotherapy, and radiation to primary tumor.

### Functional prediction of CD155 rs1058402G > *A (A67T)*

**Figure 3:**
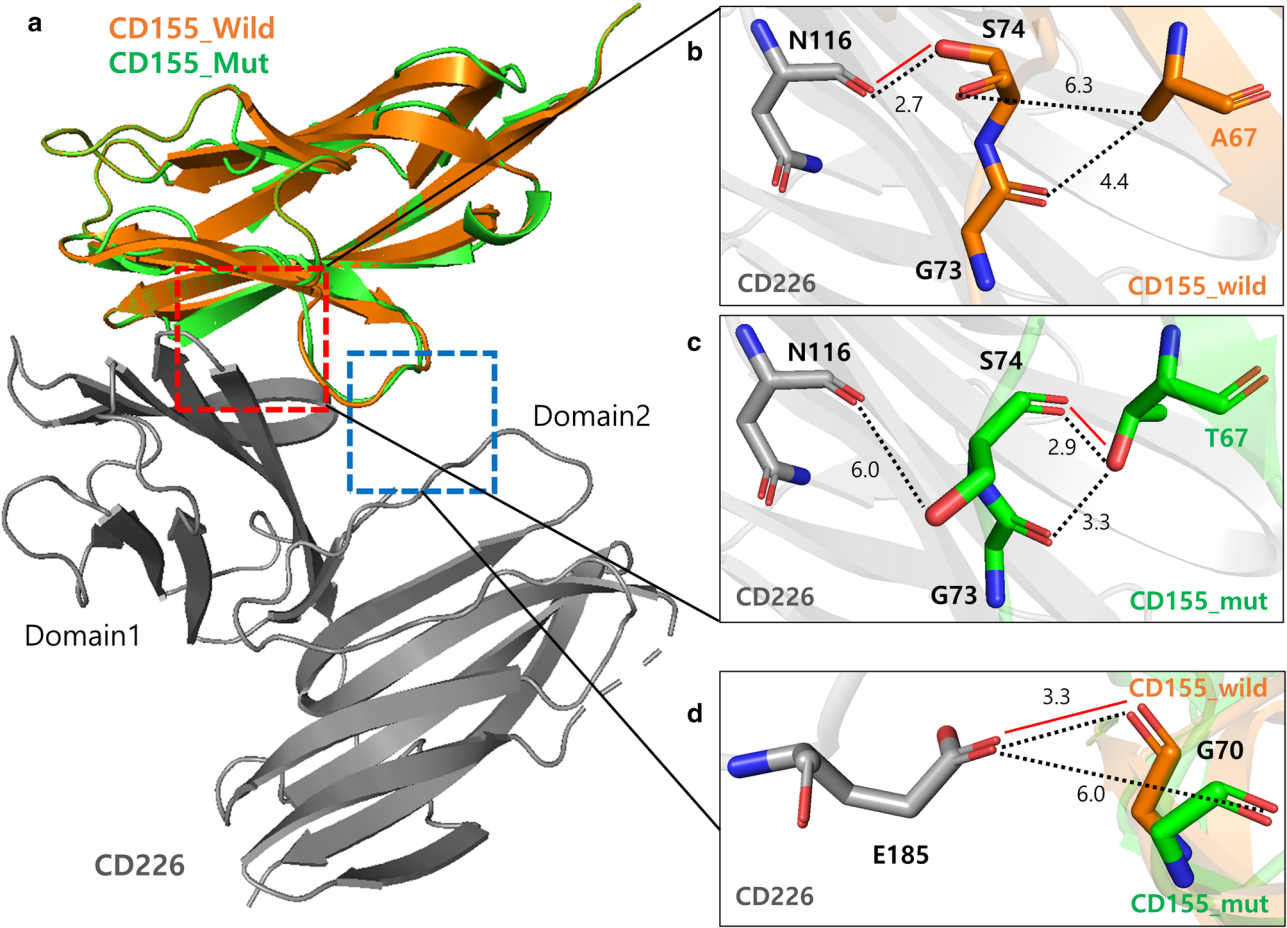
Structural model of *CD226*/*CD155* (wild-type or A67T mutant) complex. (**A**) 3-D structure of the m*CD226*-ecto (Gray) and h*CD155*-Domain1 (Orange) structures (PDB ID: 6ISC) aligned with the structural model of h*CD155*-T67 (Green). (**B**,**C**) Structural detail of the interaction between *CD226*-Domain1-N116 and *CD155*-G73, S74, A67/T67. (**D**) Structural detail of the interaction between *CD226*-Domain2-E185 and *CD155*-wild-type/mutant model G70. The related amino acid residues are shown as sticks (nitrogen atoms: blue, oxygen atoms: red). Inter-atomic distance (in Angstrom) is shown as dotted black lines. The putative polar contacts are shown as red lines. All images of *CD226*/*CD155* (wild-type vs. A67T mutant) were made using PyMOL (https://pymol.org/2/).

The rs1058402G > A changes the amino acid of alanine to threonine at codon 67 of *CD155*. *CD155* bound to *CD226* of the immune cell and acts as co-stimulatory signal^19^. We evaluated whether this amino acid change affects the function of *CD155* by creating a 3-D structural model using PyMOL (https://pymol.org/2/). As shown in Fig. 3, the alanine to threonine change at codon 67 provides distance shortening to S74 and G73 of *CD155* (6.3→2.9 and 4.4→3.3 Å, respectively; Fig. 3B,C). The shortened distance between T67 and S74 created a new hydrogen bond (Fig. 3C). This change resulted in distance lengthening between S74 of *CD155* and N116 of *CD226* (2.7→6.0 Å) and losing the hydrogen bond (Fig. 3B,C). In addition, *CD155* A67T also influenced other important interactions between G70 of *CD155* and E185 of *CD226* by distance extension (3.3→6.0 Å), resulting in the loss of the hydrogen bond (Fig. 3D). The increased distances and loss of hydrogen bonds will weaken the binding activity between *CD155* and *CD226*.

## Discussion

The immune system is evidently important not only in the development and progression of cancer but also in its treatment^4,20^. The interaction between tumors and their microenvironment (including immune cells such as tumor infiltrating lymphocytes) can affect chemotherapy response^20–22^. Genetic variants in immune genes can also influence the host’s immune activity, which affects the clinical cancer treatment outcome^12,13,23,24^. In this study, we investigated the association between variants in immune checkpoint genes and the clinical outcome of SCLC. This is the first study to investigate the effects of genetic variants in immune checkpoint genes on chemotherapy response and prognosis in SCLC. We found that two variants, namely *CD155* rs1058402G > A (A67T) and *CD226* rs763361C > T (G307S), were significantly associated with chemotherapy response and survival outcomes.

The development of drugs to block immune checkpoints has opened a new era in cancer treatment. Programmed death 1 (PD-1) and PD-L1 inhibitors are used actively in NSCLC treatment^10,11^. The PD-1/PD-L1 interaction provides inhibitory signals to suppress immune cell responses, so blocking the PD-1/PD-L1 pathway can reactivate immune cells to attack cancer. In NSCLC, the polymorphisms in PD-L1 were reported to affect chemotherapy/immunotherapy response and prognosis^12–14^. However, data on the association between polymorphisms in PD-1/PD-L1 and clinical outcomes in SCLC are limited. In this study, the polymorphisms in PD-1/PD-L1 were not associated with clinical outcomes in SCLC patients (Supplementary Table S1).

*CD155*, also called PVR or Necl-5, encodes a transmembrane glycoprotein belonging to the immunoglobulin superfamily. *CD155* was initially known to play roles in cell adhesion and migration, but recently, its roles in immunology and oncology have been noted^25,26^. *CD226* encodes a glycoprotein expressed on the surface of natural killer (NK) cells, T cells, a subset of B cells, and monocytes, and plays an important role in their activation and inhibition^27,28^. *CD155* on antigen-presenting cells or tumor cells binds to *CD226* on T cells and NK cells, which is similar to the interaction between PD-1 and PD-L1^19,29^. The difference is that PD-1/PD-L1 interaction provides an inhibitory signal to suppress T cell response, but *CD155*/*CD226* binding is a co-stimulatory interaction for T cell or NK cell activation^19,30^. In cancer immunology, *CD155* overexpression was reported in several types of human malignancies, including lung adenocarcinoma, and was correlated with unfavorable prognosis^31–33^. *CD226* has been reported to be involved in anti-tumor response by regulating NK cells^34,35^.

The rs1058402G > A is a missense mutation. The rs1058402G > A changes alanine amino acid to threonine at codon 67 of *CD155*. 3-D structure model showed that changing alanine to threonine at codon 67 shortened the distance to S74/G73 of *CD155*. The shortened distance made a new hydrogen bond between T67 and S74 of *CD155*, and this change resulted in distance extension and loss of hydrogen bond between the S74 of *CD155* and N116 of *CD226*. In addition, the structure modification due to rs1058402G > A (A67T) increased the coupling distance between the G70 of CD 155 and E185 of *CD226*, and resulted in hydrogen bond loss. In the interaction between *CD155* and *CD226*, the S74/G70 of *CD155* and N116/E185 of *CD226* are important binding points^19^. In this study, the 1058402G > A (A67T) was significantly associated with worse chemotherapy response and OS in SCLC patients. As shown in the 3-D structural model, the 1058402G > A (A67T) increased the binding point distance between *CD155* and *CD226*. The increased distance and loss of hydrogen bonds lead to a weakening of the binding force of *CD155*/*CD226*, thereby reducing the co-stimulatory signal to immune cells. The decreased immune response would had a negative effect on SCLC clinical outcomes. However, further functional studies are needed to determine whether *CD155* rs1058402G > A (A67T) affects the *CD155*/*CD226* interaction. This is the first review to report that a *CD155* variant was associated with cancer clinical outcomes. This variant may also affect the therapeutic effect of the drug to be developed in the future, considering that *CD155* is being studied as a new therapeutic target in tumor immunology^26,36^.

In the present study, *CD226* rs763361C > T was associated with better chemotherapy response and OS in SCLC patients. The rs763361C > T is located in exon 7 encoding the cytoplasmic tail of *CD226*, which harbors two phosphorylation sites and is a non-synonymous mutation^37^. The rs763361 C-to-T change results in the replacement of glycine to serine at codon 307. A Gly307Ser substitution believed to affect *CD226* expression by changing the phosphorylation or altering RNA splicing by disrupting the exon-splicing silencer sequence^37,38^. *CD226* rs763361C > T is associated with multiple autoimmune diseases, including multiple sclerosis and rheumatoid arthritis^37–40^. Regarding cancer, the T allele of rs763361 has been reported to increase the risk of NSCLC in the Chinese Han population^41^. However, the exact biological function of *CD226* rs763361C > T is not well-known, and further researches are needed. The effect on clinical outcomes may be due to other causal variants in LD. The *CD226* rs1790947G > T has a strong LD (r^2^ = 0.91) with *CD226* rs763361C > T and is located in the 3′ untranslated region. Using RegulomDB (http://www.regulomedb.org/), the rs1790947G > T was likely expected to affect binding and linked to *CD226* expression. RegulomeDB is a novel approach and database, which provides interpretation of regulatory variants in the human genome^42^. The association patterns between the rs1790947 genotypes and chemotherapy response and OS were similar to that of rs763361 (Supplementary Table S5). Hence, the selected polymorphism was rs763361C > T, but rs1790947G > T could actually affect *CD226* expression and clinical outcomes.

In this study, polymorphisms in the CD155 and CD226 was associated with survival outcomes in SCLC patients who received chemotherapy. CD155 and CD226 are one of targets in immuno-oncology^43,44^. Therefore, variants in CD155 and CD226 may have a greater impact on clinical outcomes with immunotherapy alone or in combination with chemotherapy than with chemotherapy alone. Recently, immunotherapy has been shown to be effective in the treatment of SCLC^45^. The addition of atezolizumab to etoposide/carboplatin in the first-line treatment of extensive-stage SCLC showed significant longer OS and PFS than chemotherapy alone^46^. Because CD155 and CD226 are co-stimulatory signal for immune cells, investigating the role of variants in CD155 and CD226 in SCLC patients who received combination immunotherapy and chemotherapy may yield interesting results.

Although a theoretical model for the interactional changes caused by mutations of CD155 and CD226 has been presented, one of limitations is that it has not been confirmed experimentally. Unlike NSCLC, surgical treatment is not usually performed in patients with SCLC. The difficulty of obtaining sufficient tumor tissues for experiments is an obstacle to overcome in SCLC studies. Observational retrospective study at a single center can be another limitation of this study.

In summary, we investigated the association between the variants in immune checkpoint genes and SCLC prognosis. Two variants, namely *CD155* rs1058402G > A (A67T) and *CD226* rs763361C > T (G307S), were associated with chemotherapy response and OS outcomes. *CD155* rs1058402G > A (A67T) seems to influence the interaction between *CD155* and *CD226*. However, further studies are warranted to confirm this finding.

## Supplementary information

Supplementary Information
