# Supplementary material for: Impact of immune checkpoint gene CD155 Ala67Thr and CD226 Gly307Ser polymorphisms on small cell lung cancer clinical outcome
Source: Sci Rep. 2021 Jan 19;11:1794. doi: 10.1038/s41598-021-81260-1 (PMC7815735; doi:10.1038/s41598-021-81260-1)
Supplement: Supplementary file 1 — Supplementary Information [file 41598_2021_81260_MOESM1_ESM.docx]

**Impact of immune checkpoint gene *CD155* Ala67Thr and *CD226* Gly307Ser polymorphisms on small cell lung cancer clinical outcome**

Jang Hyuck Lee^1,2^, Seung Soo Yoo^3^, Mi Jeong Hong^1,4^, Jin Eun Choi^1,4^, Soyoun Kim^1,4^, Hyo-Gyoung Kang^1,4^, Sook Kyung Do^1,5^, Ji Hyun Kim^1,4^, Sun Ah Baek^4^, Won Kee Lee^6^, Jae Do Yoo^1,2^, Sun Ha Choi^3^, Yong Hoon Lee^3^, Hyewon Seo^3^, Jaehee Lee^3^, Shin Yup Lee^3^, Seung Ick Cha^3^, Chang Ho Kim^3^ & Jae Yong Park^1,2,3,4^

^1^Department of Biochemistry and Cell Biology, School of Medicine, Kyungpook National University, Daegu, Republic of Korea; ^2^BK21 Plus KNU Biomedical Convergence Program, Department of Biomedical Science, Kyungpook National University, Daegu, Republic of Korea; ^3^Department of Internal Medicine, School of Medicine, Kyungpook National University, Kyungpook National University Hospital, Daegu, Republic of Korea; ^4^Cell and Matrix Research Institute, School of Medicine, Kyungpook National University, Daegu, Republic of Korea; ^5^Tumor Heterogeneity and Network (THEN) Research Center, School of Medicine, Kyungpook National University, Daegu, Republic of Korea; ^6^Biostatistics, Medical Research Collaboration Center, Kyungpook National University, Daegu, Republic of Korea.

Address for correspondence:

Dr. Seung Soo Yoo, Departments of Internal Medicine, School of Medicine, Kyungpook National University Chilgok Hospital, 807, Hoguk-ro, Buk-gu, Daegu 41404, Republic of Korea

Tel: 82-53-200-2631; Fax: 82-53-200-2027; E-mail: yooss@knu.ac.kr

Dr. Jae Yong Park, Departments of Internal Medicine, School of Medicine, Kyungpook National University Chilgok Hospital, 807, Hoguk-ro, Buk-gu, Daegu 41404, Republic of Korea

Tel: 82-53-200-2631; Fax: 82-53-200-2027; E-mail: jaeyong@knu.ac.kr

| Supplement Table 1. List of analyzed single nucleotide polymorphisms | | | | | | | | | | | | | | | | | | | | |
| --- | --- | --- | --- | --- | --- | --- | --- | --- | --- | --- | --- | --- | --- | --- | --- | --- | --- | --- | --- | --- |
|  |  |  |  |  |  |  |  | Chemotherapy response | | | | | |  | Overall survival | | | | | |
|  |  |  |  |  |  |  |  | Dominant | | Recessive | | Codominant | |  | Dominant | | Recessive | | Codominant | |
| SNP | Gene | 1/1^a^ | 1/2^a^ | 2/2^a^ | MAF | HWE *P* |  | *P*^b^ | OR(95%CI)^b^ | *P*^b^ | OR(95%CI)^b^ | *P*^b^ | OR(95%CI)^b^ |  | *P*^c^ | HR(95%CI)^c^ | *P*^c^ | HR(95%CI)^c^ | *P*^c^ | HR(95%CI)^c^ |
| rs3766377 | 2B4 | 106 | 108 | 44 | 0.38 | 0.07 |  | 0.75 | 1.10(0.61-1.99) | 0.09 | 2.28(0.87-5.97) | 0.28 | 1.26(0.83-1.93) |  | 0.80 | 1.04(0.78-1.38) | 0.15 | 1.34(0.90-2.01) | 0.38 | 1.10(0.89-1.35) |
| rs480104 | 2B4 | 90 | 130 | 41 | 0.41 | 0.60 |  | 0.79 | 1.09(0.58-2.03) | 0.45 | 1.38(0.60-3.20) | 0.56 | 1.14(0.74-1.76) |  | 0.04 | 0.74(0.54-0.99) | 0.53 | 0.88(0.59-1.31) | 0.08 | 0.83(0.66-1.03) |
| rs485618 | 2B4 | 158 | 85 | 11 | 0.21 | 0.92 |  | 0.14 | 0.63(0.35-1.16) | 0.12 | 0.36(0.10-1.30) | 0.07 | 0.63(0.38-1.04) |  | 0.35 | 1.15(0.86-1.55) | 0.11 | 1.75(0.88-3.48) | 0.18 | 1.19(0.92-1.53) |
| rs161810 | CD137 | 80 | 125 | 55 | 0.45 | 0.63 |  | 0.67 | 0.87(0.45-1.67) | 0.13 | 0.58(0.28-1.18) | 0.26 | 0.78(0.51-1.19) |  | 0.40 | 1.15(0.83-1.57) | 0.01 | 1.63(1.12-2.38) | 0.05 | 1.24(1.00-1.55) |
| rs348337 | CD137L | 156 | 91 | 14 | 0.23 | 0.88 |  | 0.90 | 0.96(0.53-1.74) | 0.15 | 0.43(0.13-1.36) | 0.51 | 0.85(0.53-1.37) |  | 0.49 | 1.11(0.83-1.49) | 0.06 | 1.72(0.97-3.07) | 0.21 | 1.16(0.92-1.48) |
| rs348389 | CD137L | 200 | 57 | 4 | 0.12 | 0.98 |  | 0.20 | 1.60(0.77-3.32) | 0.98 | - | 0.13 | 1.68(0.85-3.32) |  | 0.43 | 0.88(0.64-1.21) | 0.60 | 0.73(0.22-2.36) | 0.39 | 0.88(0.66-1.18) |
| rs3865469 | CD137L | 121 | 114 | 26 | 0.32 | 0.91 |  | 0.75 | 1.10(0.61-1.98) | 0.77 | 1.16(0.45-3.01) | 0.70 | 1.09(0.70-1.69) |  | 0.25 | 0.85(0.64-1.13) | 0.49 | 1.17(0.75-1.83) | 0.58 | 0.94(0.76-1.17) |
| rs1058402 | CD155 | 180 | 66 | 10 | 0.17 | 0.21 |  | 0.05 | 0.52(0.27-0.99) | 0.64 | 0.71(0.16-3.09) | 0.07 | 0.61(0.36-1.04) |  | 0.01 | 1.55(1.12-2.14) | 0.45 | 1.33(0.64-2.76) | 0.01 | 1.39(1.07-1.80) |
| rs7255066 | CD155 | 67 | 127 | 65 | 0.50 | 0.76 |  | 0.95 | 0.98(0.50-1.93) | 0.95 | 0.98(0.49-1.95) | 0.94 | 0.98(0.65-1.49) |  | 0.32 | 1.18(0.85-1.63) | 0.01 | 1.57(1.13-2.17) | 0.03 | 1.26(1.03-1.54) |
| rs9728526 | CD160 | 151 | 91 | 19 | 0.25 | 0.31 |  | 0.76 | 1.10(0.61-1.98) | 0.50 | 1.52(0.45-5.10) | 0.60 | 1.13(0.71-1.81) |  | 0.67 | 0.94(0.71-1.25) | 0.98 | 1.01(0.60-1.71) | 0.75 | 0.96(0.77-1.21) |
| rs727088 | CD226 | 140 | 99 | 19 | 0.27 | 0.80 |  | 0.10 | 1.67(0.91-3.08) | 0.59 | 0.74(0.26-2.17) | 0.29 | 1.30(0.80-2.12) |  | 0.08 | 0.77(0.58-1.04) | 0.73 | 1.10(0.63-1.91) | 0.20 | 0.85(0.67-1.09) |
| rs763361 | CD226 | 97 | 122 | 35 | 0.38 | 0.73 |  | 0.02 | 2.03(1.10-3.75) | 0.10 | 2.27(0.85-6.09) | 0.02 | 1.78(1.12-2.82) |  | 0.02 | 0.69(0.51-0.94) | 0.19 | 0.75(0.48-1.15) | 0.02 | 0.76(0.61-0.96) |
| rs11265493 | CD244 | 75 | 126 | 57 | 0.47 | 0.77 |  | 0.74 | 1.11(0.59-2.09) | 0.10 | 2.00(0.88-4.53) | 0.24 | 1.28(0.84-1.96) |  | 0.34 | 1.17(0.85-1.60) | 0.94 | 0.99(0.70-1.40) | 0.57 | 1.06(0.87-1.29) |
| rs6682654 | CD244 | 98 | 114 | 49 | 0.41 | 0.13 |  | 0.62 | 1.16(0.64-2.11) | 0.37 | 1.46(0.64-3.35) | 0.42 | 1.19(0.79-1.80) |  | 0.86 | 1.03(0.77-1.37) | 0.60 | 0.91(0.62-1.32) | 0.87 | 0.98(0.81-1.19) |
| rs11064195 | CD27 | 143 | 100 | 12 | 0.24 | 0.30 |  | 0.36 | 1.33(0.72-2.47) | 0.32 | 2.26(0.46-11.2) | 0.26 | 1.36(0.80-2.29) |  | 0.66 | 0.94(0.69-1.26) | 0.82 | 1.08(0.56-2.07) | 0.78 | 0.97(0.75-1.24) |
| rs11569361 | CD27 | 121 | 106 | 28 | 0.32 | 0.51 |  | 0.30 | 0.73(0.40-1.33) | 0.46 | 0.70(0.27-1.79) | 0.27 | 0.78(0.50-1.21) |  | 0.62 | 0.93(0.69-1.25) | 0.11 | 0.66(0.40-1.09) | 0.25 | 0.88(0.71-1.10) |
| rs2250246 | CD27 | 68 | 138 | 55 | 0.48 | 0.33 |  | 0.52 | 0.80(0.41-1.57) | 0.40 | 0.73(0.36-1.51) | 0.36 | 0.82(0.53-1.26) |  | 0.52 | 1.11(0.80-1.54) | 0.35 | 0.84(0.58-1.21) | 0.89 | 0.99(0.80-1.21) |
| rs3136551 | CD27 | 211 | 45 | 4 | 0.10 | 0.38 |  | 0.56 | 0.80(0.38-1.69) | 0.19 | 0.19(0.02-2.31) | 0.37 | 0.74(0.38-1.43) |  | 0.05 | 1.43(1.00-2.05) | 0.39 | 0.53(0.13-2.23) | 0.17 | 1.24(0.91-1.69) |
| rs2291012 | CD276 | 207 | 52 | 2 | 0.11 | 1.00 |  | 0.06 | 0.52(0.26-1.03) | 0.99 | - | 0.13 | 0.60(0.32-1.15) |  | 0.34 | 1.19(0.83-1.71) | 0.85 | 1.15(0.28-4.72) | 0.35 | 1.17(0.84-1.63) |
| rs3816661 | CD276 | 84 | 128 | 48 | 0.43 | 0.95 |  | 0.05 | 1.86(1.01-3.42) | 0.65 | 1.21(0.54-2.70) | 0.11 | 1.43(0.93-2.22) |  | 0.86 | 1.03(0.76-1.40) | 0.06 | 1.41(0.98-2.02) | 0.26 | 1.13(0.91-1.40) |
| rs3825859 | CD276 | 222 | 39 | 0 | 0.07 | 0.19 |  | 0.02 | 0.40(0.19-0.87) | - | - | 0.02 | 0.40(0.19-0.87) |  | 0.33 | 1.23(0.81-1.86) | - | - | 0.33 | 1.23(0.81-1.86) |
| rs8032531 | CD276 | 65 | 134 | 61 | 0.49 | 0.62 |  | 0.07 | 0.50(0.23-1.07) | 0.16 | 0.62(0.32-1.21) | 0.05 | 0.64(0.42-1.00) |  | 0.31 | 0.84(0.60-1.18) | 0.58 | 1.10(0.78-1.56) | 0.78 | 0.97(0.78-1.20) |
| rs1879877 | CD28 | 99 | 122 | 39 | 0.38 | 0.89 |  | 0.55 | 1.20(0.66-2.19) | 0.68 | 1.19(0.52-2.70) | 0.53 | 1.15(0.75-1.75) |  | 0.63 | 0.93(0.70-1.24) | 0.34 | 1.23(0.80-1.87) | 0.92 | 1.01(0.81-1.26) |
| rs3181098 | CD28 | 157 | 90 | 12 | 0.22 | 0.84 |  | 0.16 | 1.56(0.84-2.88) | 0.37 | 2.07(0.42-10.13) | 0.13 | 1.50(0.89-2.55) |  | 0.56 | 1.09(0.82-1.46) | 0.94 | 0.97(0.42-2.25) | 0.62 | 1.07(0.83-1.38) |
| rs3181100 | CD28 | 183 | 72 | 5 | 0.16 | 0.49 |  | 0.36 | 0.74(0.40-1.40) | 0.41 | 0.45(0.07-2.94) | 0.29 | 0.74(0.42-1.29) |  | 0.65 | 0.93(0.68-1.28) | 0.81 | 1.12(0.45-2.78) | 0.74 | 0.95(0.72-1.27) |
| rs1535043 | CD40 | 114 | 117 | 29 | 0.34 | 0.90 |  | 0.48 | 1.24(0.68-2.25) | 0.34 | 1.67(0.58-4.88) | 0.33 | 1.26(0.79-2.00) |  | 0.79 | 0.96(0.71-1.29) | 0.65 | 0.90(0.56-1.43) | 0.67 | 0.95(0.76-1.19) |
| rs1883832 | CD40 | 117 | 118 | 26 | 0.33 | 0.64 |  | 0.33 | 0.74(0.41-1.34) | 0.94 | 0.96(0.36-2.58) | 0.43 | 0.84(0.53-1.31) |  | 0.40 | 1.13(0.85-1.51) | 0.77 | 0.92(0.55-1.56) | 0.60 | 1.06(0.85-1.33) |
| rs4813003 | CD40 | 122 | 105 | 32 | 0.33 | 0.21 |  | 0.41 | 0.78(0.43-1.41) | 0.95 | 0.97(0.39-2.42) | 0.53 | 0.87(0.57-1.34) |  | 0.39 | 1.13(0.85-1.51) | 0.56 | 0.87(0.55-1.39) | 0.72 | 1.04(0.84-1.28) |
| rs6032678 | CD40 | 108 | 124 | 29 | 0.35 | 0.46 |  | 0.47 | 0.80(0.44-1.47) | 0.77 | 1.16(0.43-3.17) | 0.68 | 0.91(0.58-1.43) |  | 0.62 | 1.08(0.80-1.45) | 0.61 | 0.89(0.56-1.41) | 0.90 | 1.01(0.82-1.26) |
| rs752118 | CD40 | 127 | 110 | 23 | 0.30 | 0.91 |  | 0.61 | 0.86(0.47-1.55) | 0.40 | 1.65(0.52-5.26) | 0.98 | 0.99(0.63-1.58) |  | 0.87 | 0.98(0.73-1.31) | 0.51 | 0.84(0.50-1.41) | 0.67 | 0.95(0.76-1.19) |
| rs162066 | CD47 | 156 | 93 | 12 | 0.22 | 0.69 |  | 0.93 | 0.98(0.54-1.77) | 0.33 | 2.22(0.45-10.87) | 0.76 | 1.08(0.66-1.78) |  | 0.00 | 0.65(0.48-0.87) | 0.66 | 0.86(0.44-1.67) | 0.01 | 0.72(0.56-0.93) |
| rs326341 | CD47 | 100 | 125 | 34 | 0.37 | 0.60 |  | 0.42 | 0.78(0.42-1.43) | 0.10 | 2.39(0.85-6.72) | 0.80 | 1.06(0.68-1.64) |  | 0.00 | 0.64(0.48-0.85) | 0.49 | 0.85(0.53-1.36) | 0.01 | 0.73(0.58-0.92) |
| rs3762681 | CD47 | 190 | 65 | 6 | 0.15 | 0.87 |  | 0.92 | 1.03(0.53-2.01) | 0.48 | 2.25(0.24-21.05) | 0.75 | 1.10(0.61-1.98) |  | 0.80 | 0.96(0.68-1.35) | 0.84 | 1.11(0.40-3.06) | 0.86 | 0.97(0.72-1.32) |
| rs3828376 | CD47 | 82 | 125 | 51 | 0.44 | 0.79 |  | 0.75 | 1.11(0.59-2.08) | 0.74 | 1.14(0.53-2.42) | 0.69 | 1.09(0.72-1.65) |  | 0.05 | 0.74(0.55-1.00) | 0.42 | 0.86(0.59-1.25) | 0.09 | 0.84(0.68-1.03) |
| rs9879947 | CD47 | 142 | 100 | 19 | 0.26 | 0.81 |  | 0.83 | 1.07(0.59-1.92) | 0.93 | 0.95(0.31-2.93) | 0.89 | 1.03(0.65-1.65) |  | 0.08 | 0.78(0.58-1.03) | 0.09 | 0.60(0.34-1.08) | 0.04 | 0.79(0.63-0.98) |
| rs344590 | CD70 | 223 | 35 | 3 | 0.08 | 0.23 |  | 0.76 | 1.14(0.48-2.69) | 0.22 | 0.20(0.02-2.54) | 0.90 | 0.96(0.46-1.99) |  | 0.62 | 1.11(0.74-1.67) | 0.82 | 0.84(0.19-3.64) | 0.71 | 1.07(0.74-1.55) |
| rs344591 | CD70 | 148 | 99 | 13 | 0.24 | 0.49 |  | 0.40 | 0.78(0.43-1.40) | 0.58 | 0.69(0.19-2.54) | 0.37 | 0.80(0.49-1.30) |  | 0.53 | 0.91(0.68-1.22) | 0.86 | 0.95(0.52-1.74) | 0.55 | 0.93(0.73-1.19) |
| rs1599796 | CD80 | 140 | 94 | 27 | 0.28 | 0.07 |  | 0.95 | 1.02(0.57-1.83) | 0.90 | 1.07(0.41-2.78) | 0.91 | 1.02(0.67-1.58) |  | 0.88 | 1.02(0.76-1.37) | 0.53 | 0.86(0.53-1.39) | 0.86 | 0.98(0.79-1.22) |
| rs16829984 | CD80 | 99 | 118 | 44 | 0.39 | 0.39 |  | 0.30 | 0.72(0.39-1.34) | 0.46 | 0.75(0.35-1.60) | 0.27 | 0.79(0.52-1.20) |  | 0.56 | 1.09(0.82-1.46) | 0.49 | 1.15(0.78-1.68) | 0.44 | 1.08(0.88-1.33) |
| rs17281703 | CD80 | 209 | 48 | 4 | 0.11 | 0.52 |  | 0.86 | 1.07(0.51-2.26) | 0.28 | 0.33(0.04-2.51) | 0.87 | 0.95(0.49-1.81) |  | 0.72 | 1.07(0.74-1.55) | 0.29 | 0.46(0.11-1.95) | 0.98 | 1.00(0.72-1.38) |
| rs1880661 | CD80 | 156 | 86 | 19 | 0.24 | 0.14 |  | 0.89 | 1.04(0.57-1.90) | 0.95 | 1.04(0.34-3.17) | 0.89 | 1.03(0.65-1.64) |  | 0.46 | 1.11(0.84-1.48) | 0.57 | 0.85(0.50-1.47) | 0.76 | 1.03(0.84-1.28) |
| rs2049502 | CD80 | 115 | 112 | 33 | 0.34 | 0.49 |  | 0.35 | 0.75(0.41-1.37) | 0.35 | 1.56(0.62-3.94) | 0.83 | 0.96(0.63-1.46) |  | 0.09 | 1.29(0.96-1.73) | 0.50 | 1.15(0.76-1.74) | 0.12 | 1.18(0.96-1.44) |
| rs2222791 | CD80 | 171 | 78 | 10 | 0.19 | 0.77 |  | 0.70 | 0.89(0.48-1.63) | 0.24 | 3.56(0.43-29.38) | 0.91 | 1.03(0.61-1.74) |  | 0.30 | 0.86(0.64-1.15) | 0.35 | 0.72(0.36-1.44) | 0.23 | 0.86(0.67-1.10) |
| rs7628626 | CD80 | 189 | 68 | 4 | 0.15 | 0.45 |  | 0.82 | 0.93(0.48-1.79) | 0.87 | 0.82(0.08-8.28) | 0.80 | 0.93(0.51-1.69) |  | 0.99 | 1.00(0.73-1.37) | 0.95 | 0.96(0.30-3.07) | 0.98 | 1.00(0.74-1.34) |
| rs13082681 | CD86 | 185 | 71 | 5 | 0.16 | 0.54 |  | 0.54 | 0.82(0.44-1.55) | 0.07 | 0.17(0.03-1.12) | 0.27 | 0.73(0.42-1.28) |  | 0.14 | 0.78(0.57-1.08) | 0.18 | 1.87(0.74-4.70) | 0.29 | 0.85(0.63-1.15) |
| rs4478030 | CD86 | 130 | 114 | 17 | 0.28 | 0.23 |  | 0.98 | 0.99(0.55-1.79) | 0.72 | 1.24(0.37-4.14) | 0.90 | 1.03(0.64-1.67) |  | 0.28 | 1.17(0.88-1.57) | 0.28 | 0.71(0.39-1.32) | 0.70 | 1.05(0.84-1.31) |
| rs11666350 | CEACAM1 | 163 | 84 | 14 | 0.21 | 0.47 |  | 0.08 | 0.58(0.32-1.08) | 0.33 | 0.54(0.16-1.85) | 0.08 | 0.64(0.39-1.05) |  | 0.28 | 0.85(0.62-1.15) | 0.54 | 0.83(0.46-1.50) | 0.27 | 0.87(0.68-1.11) |
| rs8102519 | CEACAM1 | 220 | 39 | 2 | 0.08 | 0.85 |  | 0.12 | 0.54(0.25-1.17) | 0.28 | 0.18(0.01-3.89) | 0.09 | 0.53(0.26-1.09) |  | 0.74 | 0.93(0.63-1.39) | 0.53 | 1.60(0.37-7.01) | 0.85 | 0.97(0.66-1.41) |
| rs11571316 | CTLA4 | 188 | 67 | 6 | 0.15 | 0.99 |  | 0.31 | 1.41(0.72-2.77) | 0.56 | 1.94(0.21-17.59) | 0.29 | 1.39(0.76-2.53) |  | 0.15 | 1.27(0.92-1.76) | 0.43 | 0.62(0.19-2.01) | 0.32 | 1.16(0.87-1.54) |
| rs231775 | CTLA4 | 135 | 103 | 21 | 0.28 | 0.83 |  | 0.16 | 0.66(0.36-1.18) | 0.98 | 1.01(0.33-3.07) | 0.27 | 0.78(0.49-1.22) |  | 0.26 | 1.18(0.89-1.56) | 0.67 | 1.13(0.65-1.98) | 0.28 | 1.13(0.90-1.42) |
| rs5742909 | CTLA4 | 199 | 58 | 3 | 0.12 | 0.59 |  | 0.04 | 0.50(0.26-0.98) | 0.07 | 0.08(0.01-1.24) | 0.02 | 0.48(0.26-0.89) |  | 0.84 | 0.97(0.69-1.35) | 0.18 | 2.29(0.69-7.64) | 0.95 | 1.01(0.74-1.38) |
| rs733618 | CTLA4 | 76 | 129 | 56 | 0.46 | 0.93 |  | 0.57 | 1.20(0.64-2.23) | 0.45 | 1.32(0.64-2.73) | 0.43 | 1.18(0.79-1.76) |  | 0.48 | 1.12(0.82-1.52) | 0.98 | 1.00(0.71-1.41) | 0.65 | 1.05(0.86-1.27) |
| rs3763959 | galectin9 | 125 | 111 | 24 | 0.31 | 0.93 |  | 0.29 | 0.73(0.41-1.31) | 0.69 | 0.82(0.30-2.20) | 0.32 | 0.80(0.51-1.25) |  | 0.33 | 0.87(0.65-1.15) | 0.62 | 0.88(0.54-1.45) | 0.33 | 0.90(0.72-1.12) |
| rs3819001 | GITR | 208 | 45 | 6 | 0.11 | 0.07 |  | 0.70 | 0.87(0.43-1.77) | 0.62 | 0.63(0.11-3.84) | 0.62 | 0.86(0.47-1.57) |  | 0.23 | 1.25(0.87-1.79) | 0.52 | 1.32(0.56-3.13) | 0.22 | 1.21(0.90-1.63) |
| rs10158166 | H7B4 | 166 | 85 | 10 | 0.20 | 0.83 |  | 0.65 | 1.16(0.62-2.19) | 0.83 | 1.19(0.23-6.24) | 0.64 | 1.14(0.66-1.98) |  | 0.97 | 1.01(0.74-1.36) | 0.21 | 0.59(0.26-1.35) | 0.66 | 0.94(0.73-1.22) |
| rs10754339 | H7B4 | 203 | 55 | 3 | 0.12 | 0.74 |  | 0.38 | 1.38(0.68-2.80) | 0.90 | 0.86(0.07-10.06) | 0.43 | 1.30(0.68-2.50) |  | 0.94 | 1.01(0.72-1.43) | 0.15 | 0.42(0.13-1.37) | 0.64 | 0.93(0.69-1.25) |
| rs3738414 | H7B4 | 155 | 91 | 14 | 0.23 | 0.89 |  | 0.77 | 1.10(0.59-2.03) | 0.33 | 0.54(0.15-1.89) | 0.90 | 0.97(0.59-1.60) |  | 0.23 | 1.21(0.89-1.63) | 0.72 | 0.88(0.44-1.77) | 0.39 | 1.11(0.87-1.42) |
| rs1886730 | HVEM | 76 | 116 | 67 | 0.48 | 0.10 |  | 0.39 | 1.33(0.69-2.56) | 0.72 | 0.88(0.45-1.73) | 0.76 | 1.07(0.71-1.60) |  | 0.47 | 0.89(0.64-1.23) | 0.62 | 0.92(0.67-1.27) | 0.47 | 0.93(0.77-1.13) |
| rs3762440 | HVEM | 146 | 94 | 21 | 0.26 | 0.29 |  | 0.21 | 0.69(0.39-1.23) | 0.82 | 0.88(0.29-2.70) | 0.28 | 0.78(0.49-1.23) |  | 0.23 | 1.19(0.90-1.58) | 0.29 | 0.74(0.42-1.29) | 0.65 | 1.05(0.85-1.30) |
| rs10183087 | ICOS | 193 | 55 | 7 | 0.14 | 0.21 |  | 0.23 | 1.58(0.75-3.32) | 0.91 | 1.10(0.20-6.17) | 0.30 | 1.39(0.75-2.60) |  | 0.68 | 1.07(0.77-1.50) | 0.86 | 0.93(0.39-2.18) | 0.78 | 1.04(0.79-1.37) |
| rs10932029 | ICOS | 223 | 36 | 2 | 0.08 | 0.68 |  | 0.59 | 1.25(0.56-2.83) | 0.99 | - | 0.49 | 1.31(0.60-2.84) |  | 0.89 | 1.03(0.70-1.51) | 0.12 | 3.11(0.74-13.17) | 0.70 | 1.07(0.75-1.55) |
| rs11883722 | ICOS | 57 | 130 | 74 | 0.47 | 0.99 |  | 0.66 | 1.17(0.59-2.32) | 0.15 | 0.63(0.33-1.19) | 0.52 | 0.87(0.58-1.32) |  | 0.09 | 0.74(0.52-1.05) | 0.70 | 1.06(0.78-1.46) | 0.48 | 0.93(0.75-1.15) |
| rs4335928 | ICOS | 200 | 57 | 4 | 0.12 | 0.98 |  | 0.55 | 0.82(0.42-1.59) | 0.24 | 0.29(0.04-2.30) | 0.39 | 0.77(0.42-1.40) |  | 0.35 | 0.85(0.61-1.19) | 0.28 | 0.53(0.17-1.69) | 0.24 | 0.84(0.62-1.13) |
| rs4452124 | ICOS | 98 | 113 | 50 | 0.41 | 0.09 |  | 0.83 | 1.07(0.59-1.94) | 0.98 | 0.99(0.47-2.11) | 0.90 | 1.03(0.69-1.53) |  | 0.86 | 1.03(0.76-1.39) | 0.55 | 0.90(0.62-1.29) | 0.83 | 0.98(0.81-1.19) |
| rs15927 | ICOS_L | 73 | 123 | 65 | 0.48 | 0.36 |  | 0.18 | 1.55(0.82-2.93) | 0.79 | 1.10(0.55-2.20) | 0.32 | 1.23(0.82-1.86) |  | 0.42 | 0.88(0.64-1.21) | 0.78 | 0.95(0.68-1.33) | 0.52 | 0.94(0.77-1.14) |
| rs378299 | ICOS_L | 116 | 120 | 23 | 0.32 | 0.30 |  | 0.74 | 0.90(0.50-1.63) | 0.99 | 1.01(0.36-2.84) | 0.80 | 0.94(0.59-1.49) |  | 0.98 | 1.00(0.75-1.33) | 0.73 | 0.91(0.53-1.56) | 0.86 | 0.98(0.78-1.23) |
| rs4819388 | ICOS_L | 104 | 112 | 44 | 0.38 | 0.15 |  | 0.82 | 0.93(0.51-1.70) | 0.55 | 0.79(0.37-1.69) | 0.64 | 0.91(0.60-1.36) |  | 0.59 | 1.08(0.81-1.45) | 0.94 | 1.02(0.69-1.49) | 0.68 | 1.04(0.86-1.27) |
| rs3824259 | IDO | 75 | 126 | 59 | 0.47 | 0.66 |  | 0.23 | 1.48(0.78-2.78) | 0.71 | 0.88(0.44-1.75) | 0.58 | 1.12(0.74-1.69) |  | 0.68 | 0.94(0.68-1.29) | 0.41 | 1.15(0.82-1.63) | 0.83 | 1.02(0.83-1.26) |
| rs870849 | LAG3 | 174 | 73 | 8 | 0.17 | 0.92 |  | 0.34 | 0.73(0.38-1.40) | 0.48 | 0.53(0.09-3.05) | 0.29 | 0.74(0.42-1.30) |  | 0.76 | 1.05(0.77-1.44) | 0.53 | 0.75(0.30-1.86) | 0.96 | 1.01(0.77-1.32) |
| rs11878563 | LIGHT | 63 | 143 | 55 | 0.48 | 0.12 |  | 0.65 | 1.17(0.59-2.32) | 0.31 | 0.70(0.35-1.40) | 0.74 | 0.93(0.60-1.44) |  | 0.92 | 1.02(0.71-1.46) | 0.18 | 0.79(0.56-1.12) | 0.42 | 0.92(0.74-1.13) |
| rs344560 | LIGHT | 224 | 35 | 2 | 0.07 | 0.63 |  | 0.64 | 1.22(0.52-2.85) | 0.54 | 0.41(0.02-7.03) | 0.79 | 1.11(0.52-2.40) |  | 1.00 | 1.00(0.66-1.51) | 0.19 | 2.67(0.62-11.49) | 0.81 | 1.05(0.71-1.55) |
| rs12140760 | OX40L | 103 | 122 | 35 | 0.37 | 0.91 |  | 0.25 | 0.69(0.38-1.29) | 0.13 | 0.52(0.22-1.21) | 0.11 | 0.69(0.44-1.08) |  | 0.50 | 1.11(0.82-1.49) | 0.14 | 1.38(0.90-2.12) | 0.22 | 1.15(0.92-1.44) |
| rs1234315 | OX40L | 97 | 128 | 33 | 0.38 | 0.36 |  | 0.37 | 0.76(0.41-1.39) | 0.89 | 1.06(0.44-2.58) | 0.55 | 0.88(0.57-1.36) |  | 0.36 | 1.14(0.86-1.52) | 0.41 | 0.84(0.55-1.28) | 0.82 | 1.02(0.84-1.25) |
| rs1539259 | OX40L | 68 | 132 | 61 | 0.49 | 0.84 |  | 0.24 | 0.65(0.31-1.34) | 0.79 | 0.91(0.45-1.85) | 0.37 | 0.82(0.53-1.27) |  | 0.17 | 1.27(0.90-1.78) | 0.49 | 0.89(0.63-1.24) | 0.67 | 1.04(0.86-1.27) |
| rs16845543 | OX40L | 227 | 33 | 1 | 0.07 | 0.86 |  | 0.47 | 1.41(0.56-3.56) | 0.99 | - | 0.65 | 1.22(0.52-2.86) |  | 0.78 | 0.94(0.61-1.45) | 0.77 | 1.34(0.18-10.14) | 0.83 | 0.95(0.63-1.45) |
| rs16845679 | OX40L | 165 | 81 | 15 | 0.21 | 0.24 |  | 0.66 | 1.15(0.62-2.13) | 0.57 | 1.47(0.38-5.67) | 0.57 | 1.16(0.70-1.91) |  | 0.31 | 0.86(0.63-1.16) | 0.30 | 0.72(0.38-1.34) | 0.22 | 0.86(0.68-1.10) |
| rs3861953 | OX40L | 202 | 57 | 2 | 0.12 | 0.35 |  | 0.66 | 1.18(0.57-2.44) | 0.99 | - | 0.60 | 1.21(0.60-2.46) |  | 0.28 | 1.21(0.86-1.70) | 0.44 | 0.46(0.06-3.39) | 0.41 | 1.14(0.83-1.57) |
| rs4916215 | OX40L | 213 | 43 | 1 | 0.09 | 0.45 |  | 0.38 | 1.48(0.61-3.59) | 0.99 | - | 0.36 | 1.50(0.63-3.58) |  | 0.70 | 0.92(0.61-1.40) | 0.87 | 1.18(0.16-8.64) | 0.74 | 0.93(0.63-1.39) |
| rs6425217 | OX40L | 130 | 100 | 23 | 0.29 | 0.55 |  | 0.75 | 1.11(0.60-2.02) | 0.79 | 1.16(0.39-3.42) | 0.72 | 1.09(0.69-1.73) |  | 0.24 | 0.84(0.63-1.13) | 0.33 | 0.78(0.47-1.28) | 0.19 | 0.86(0.69-1.08) |
| rs10204525 | PD1 | 156 | 85 | 18 | 0.23 | 0.18 |  | 0.30 | 0.72(0.40-1.33) | 0.58 | 0.72(0.23-2.29) | 0.29 | 0.78(0.48-1.25) |  | 0.35 | 1.15(0.86-1.55) | 0.62 | 1.16(0.64-2.12) | 0.34 | 1.12(0.89-1.42) |
| rs2227982 | PD1 | 76 | 119 | 65 | 0.48 | 0.18 |  | 0.70 | 1.14(0.60-2.15) | 0.67 | 1.16(0.58-2.31) | 0.62 | 1.11(0.74-1.65) |  | 0.35 | 0.86(0.62-1.18) | 0.07 | 0.72(0.51-1.02) | 0.09 | 0.84(0.69-1.03) |
| rs2297136 | PDL1 | 179 | 77 | 5 | 0.17 | 0.32 |  | 0.52 | 1.23(0.65-2.34) | 0.32 | 0.38(0.06-2.58) | 0.75 | 1.10(0.62-1.97) |  | 0.74 | 1.05(0.78-1.42) | 0.01 | 3.36(1.34-8.41) | 0.41 | 1.13(0.85-1.50) |
| rs822336 | PDL1 | 137 | 103 | 19 | 0.27 | 0.95 |  | 0.56 | 0.84(0.46-1.53) | 0.35 | 0.61(0.22-1.72) | 0.39 | 0.82(0.51-1.30) |  | 0.99 | 1.00(0.74-1.35) | 0.05 | 1.74(0.99-3.03) | 0.46 | 1.10(0.86-1.41) |
| rs822337 | PDL1 | 112 | 119 | 30 | 0.34 | 0.85 |  | 0.37 | 0.76(0.42-1.38) | 0.09 | 0.47(0.20-1.11) | 0.13 | 0.71(0.46-1.11) |  | 0.85 | 1.03(0.77-1.37) | 0.00 | 2.02(1.30-3.14) | 0.13 | 1.20(0.95-1.51) |
| rs822338 | PDL1 | 84 | 129 | 47 | 0.43 | 0.84 |  | 0.43 | 0.77(0.40-1.47) | 0.15 | 0.58(0.27-1.22) | 0.18 | 0.74(0.48-1.15) |  | 0.94 | 1.01(0.74-1.38) | 0.32 | 1.20(0.84-1.74) | 0.55 | 1.07(0.86-1.32) |
| rs16923189 | PDL2 | 195 | 48 | 7 | 0.12 | 0.07 |  | 0.61 | 0.83(0.41-1.69) | 0.35 | 0.44(0.08-2.42) | 0.45 | 0.80(0.44-1.44) |  | 0.10 | 1.35(0.94-1.93) | 0.07 | 2.25(0.95-5.32) | 0.05 | 1.36(1.00-1.84) |
| rs3824448 | PDL2 | 217 | 44 | 0 | 0.08 | 0.14 |  | 0.25 | 1.65(0.70-3.85) | - | - | 0.25 | 1.65(0.70-3.85) |  | 0.43 | 0.85(0.58-1.26) | - | - | 0.43 | 0.85(0.58-1.26) |
| rs7854413 | PDL2 | 195 | 61 | 3 | 0.13 | 0.46 |  | 0.04 | 0.49(0.25-0.96) | 0.85 | 0.79(0.07-9.05) | 0.05 | 0.54(0.29-1.00) |  | 0.50 | 1.13(0.80-1.59) | 0.71 | 0.76(0.19-3.15) | 0.60 | 1.09(0.79-1.49) |
| rs1884565 | SIRPA | 114 | 111 | 36 | 0.35 | 0.29 |  | 0.56 | 1.19(0.66-2.16) | 0.17 | 1.90(0.77-4.73) | 0.26 | 1.28(0.83-1.95) |  | 0.20 | 1.22(0.90-1.64) | 0.28 | 0.80(0.53-1.20) | 0.77 | 1.03(0.84-1.26) |
| rs2235747 | SIRPA | 80 | 124 | 56 | 0.45 | 0.54 |  | 0.50 | 1.24(0.66-2.32) | 0.44 | 1.34(0.64-2.83) | 0.38 | 1.20(0.80-1.82) |  | 0.30 | 0.85(0.62-1.16) | 0.81 | 1.04(0.73-1.48) | 0.60 | 0.95(0.77-1.16) |
| rs2693052 | TIGIT | 207 | 50 | 1 | 0.10 | 0.27 |  | 0.58 | 1.24(0.58-2.67) | 0.99 | - | 0.50 | 1.29(0.62-2.70) |  | 0.00 | 1.72(1.21-2.45) | 0.79 | 1.32(0.18-9.81) | 0.00 | 1.64(1.18-2.29) |
| rs12569990 | VISTA | 138 | 108 | 15 | 0.26 | 0.30 |  | 0.37 | 0.77(0.43-1.38) | 0.26 | 2.45(0.52-11.59) | 0.77 | 0.93(0.58-1.50) |  | 0.01 | 1.50(1.13-2.00) | 0.09 | 1.68(0.92-3.08) | 0.00 | 1.42(1.13-1.80) |
| rs1867977 | VISTA | 81 | 140 | 40 | 0.42 | 0.11 |  | 0.27 | 0.70(0.36-1.33) | 0.78 | 1.12(0.50-2.55) | 0.54 | 0.87(0.56-1.35) |  | 0.07 | 1.34(0.98-1.82) | 0.78 | 1.06(0.72-1.56) | 0.15 | 1.16(0.95-1.42) |
| rs3747858 | VISTA | 73 | 140 | 47 | 0.45 | 0.16 |  | 0.68 | 1.14(0.60-2.17) | 0.52 | 0.79(0.38-1.63) | 0.92 | 0.98(0.64-1.50) |  | 0.20 | 1.23(0.89-1.70) | 0.00 | 1.69(1.18-2.43) | 0.01 | 1.32(1.06-1.65) |
| rs748503 | VISTA | 125 | 114 | 22 | 0.30 | 0.58 |  | 0.82 | 0.94(0.52-1.67) | 0.43 | 0.68(0.26-1.79) | 0.60 | 0.89(0.57-1.39) |  | 0.42 | 0.89(0.67-1.18) | 0.46 | 0.82(0.48-1.39) | 0.34 | 0.90(0.72-1.12) |
| rs748504 | VISTA | 88 | 134 | 39 | 0.41 | 0.30 |  | 0.75 | 0.90(0.48-1.69) | 0.17 | 0.57(0.26-1.27) | 0.34 | 0.81(0.52-1.25) |  | 0.07 | 0.76(0.57-1.02) | 0.71 | 0.93(0.63-1.38) | 0.15 | 0.85(0.69-1.06) |
| Abbreviation: MAF, minor allele frequency; HWE, Hard-Weinberg equilibrium; OR, odds ratio; CI, confidence interval; MST, median survival time (months); HR, hazard ratio  ^a^1 denotes a wild allele and 2 denote a polymorphic allele.  ^b^OR, 95% CI, and their corresponding *P*-values were calculated by multivariate regression analysis, adjusted for age, gender, smoking status, stage, Eastern Cooperative Oncology Group performance status, weight loss, neuron specific enolase level, and first chemotherapy regimen.  ^c^HR, 95% CI and their corresponding *P*-values were calculated using multivariate Cox proportional hazard models, adjusted for age, gender, smoking status, stage, Eastern Cooperative Oncology Group performance status, weight loss, neuron specific enolase level, first chemotherapy regimen, second line chemotherapy, and radiation to primary tumor. | | | | | | | | | | | | | | | | | | | | |

| Supplementary Table 2. In the extensive stage, chemotherapy response and survival outcomes according to *CD155* rs1058402 and *CD226* rs763361 genotypes. | | | | | | | | | | | | | | | |
| --- | --- | --- | --- | --- | --- | --- | --- | --- | --- | --- | --- | --- | --- | --- | --- |
|  |  | Chemotherapy response | | | |  | Overall survival | | | |  | Progression-free survival | | |  |
| Polymorphism | No. of case (%)^a^ | Responder  (%)^b^ | Non-responder  (%)^b^ | OR (95% CI)^c^ | *P*^c^ |  | MST (95% CI) | Log-Rank *P* | HR (95% CI)^d^ | *P*^d^ |  | Log-Rank *P* | HR (95% CI)^d^ | *P*^d^ |  |
|  |  |  |  |  |  |  |  |  |  |  |  |  |  |  |  |
| rs1058402 |  |  |  |  |  |  |  |  |  |  |  |  |  |  |  |
| GG | 133 (69.6) | 104 (78.2) | 29 (21.8) | 1.00 |  |  | 10.4 (8.8-11.6) |  | 1.00 |  |  |  | 1.00 |  |  |
| GA | 51 (26.7) | 33 (64.7) | 18 (35.3) | 0.43 (0.20-0.93) | 0.03 |  | 7.2 (5.3-8.5) |  | 1.73 (1.18-2.53) | 0.005 |  |  | 1.31 (0.91-1.89) | 0.15 |  |
| AA | 7 (3.7) | 5 (71.4) | 2 (28.6) | 0.49 (0.08-3.12) | 0.45 |  | 6.2 (2.5-10.8) | 0.09 | 1.99 (0.82-4.81) | 0.13 |  | 0.36 | 1.59 (0.68-3.75) | 0.29 |  |
| Dominant |  |  |  | 0.44 (0.21-0.92) | 0.03 |  | 8.5 (7.0-10.6) | 0.03 | 1.75 (1.21-2.53) | 0.003 |  | 0.28 | 1.34 (0.94-1.90) | 0.11 |  |
| Recessive |  |  |  | 0.68 (0.11-4.28) | 0.68 |  | 9.0 (7.6-10.4) | 0.63 | 1.62 (0.68-3.90) | 0.28 |  | 0.6 | 1.46 (0.62-3.40) | 0.39 |  |
| Codominant |  |  |  | 0.52 (0.28-0.98) | 0.04 |  |  |  | 1.58 (1.16-2.14) | 0.003 |  |  | 1.29 (0.96-1.74) | 0.09 |  |
| rs763361 |  |  |  |  |  |  |  |  |  |  |  |  |  |  |  |
| CC | 72 (37.7) | 51 (70.8) | 21 (29.2) | 1.00 |  |  | 8.5 (7.0-10.6) |  | 1.00 |  |  |  | 1.00 |  |  |
| CT | 99 (51.8) | 76 (76.8) | 23 (23.2) | 1.52 (0.72-3.21) | 0.27 |  | 9.2 (7.6-10.8) |  | 0.81 (0.56-1.16) | 0.25 |  |  | 0.80 (0.57-1.13) | 0.21 |  |
| TT | 20 (10.5) | 17 (85.0) | 3 (15.0) | 3.02 (0.74-12.27) | 0.12 |  | 13.5 (9.5-16.0) | 0.006 | 0.66 (0.36-1.23) | 0.19 |  | 0.27 | 0.69 (0.39-1.21) | 0.19 |  |
| Dominant |  |  |  | 1.69 (0.82-3.48) | 0.16 |  | 10.4 (8.7-11.6) | 0.006 | 0.79 (0.55-1.12) | 0.19 |  | 0.14 | 0.79 (0.56-1.10) | 0.16 |  |
| Recessive |  |  |  | 2.35 (0.62-8.90) | 0.21 |  | 13.5 (9.5-16.0) | 0.02 | 0.77 (0.44-1.35) | 0.36 |  | 0.29 | 0.80 (0.48-1.33) | 0.4 |  |
| Codominant |  |  |  | 1.64 (0.92-2.91) | 0.09 |  |  |  | 0.81 (0.62-1.07) | 0.14 |  |  | 0.82 (0.63-1.06) | 0.13 |  |
| Abbreviation: OR, odds ratio; CI, confidence interval; MST, median survival time (months); HR, hazard ratio.  ^a^Column percentage.  ^b^Row percentage.  ^c^OR, 95% CI, and their corresponding *P*-values were calculated by multivariate regression analysis, adjusted for age, gender, smoking status, stage, Eastern Cooperative Oncology Group performance status, weight loss, neuron specific enolase level, and first chemotherapy regimen.  ^d^HR, 95% CI and their corresponding *P*-values were calculated using multivariate Cox proportional hazard models, adjusted for age, gender, smoking status, stage, Eastern Cooperative Oncology Group performance status, weight loss, neuron specific enolase level, first chemotherapy regimen, second line chemotherapy, and radiation to primary tumor. | | | | | | | | | | | | | | | |

| Supplement Table 3. Stratified analysis of the rs763361 and rs1058402 genotypes under a dominant model | | | | | | | | | | | | | | | |
| --- | --- | --- | --- | --- | --- | --- | --- | --- | --- | --- | --- | --- | --- | --- | --- |
|  | rs1058402 | | | | | | |  | rs763361 | | | | | | |
|  | Chemotherapy response | | |  | Overall survival | | |  | Chemotherapy response | | |  | Overall survival | | |
|  | OR (95% CI)^a^ | *P*^a^ | *P*_H_ |  | HR (95% CI)^b^ | *P*^b^ | *P*_H_ |  | OR (95% CI)^a^ | *P*^a^ | *P*_H_ |  | HR (95% CI)^b^ | *P*^b^ | *P*_H_ |
| Age (year) |  |  |  |  |  |  |  |  |  |  |  |  |  |  |  |
| < 68 | 0.62(0.22-1.74) | 0.36 | 0.47 |  | 1.34(0.84-2.15) | 0.23 | 0.48 |  | 4.60(1.39-15.3) | 0.01 | 0.23 |  | 0.45(0.28-0.73) | 0.001 | 0.08 |
| ≥ 68 | 0.37(0.15-0.94) | 0.04 |  |  | 1.71(1.05-2.77) | 0.03 |  |  | 1.87(0.81-4.31) | 0.14 |  |  | 0.81(0.52-1.26) | 0.35 |  |
| Gender |  |  |  |  |  |  |  |  |  |  |  |  |  |  |  |
| Male | 0.56(0.28-1.11) | 0.10 | 0.54 |  | 1.67(1.19-2.35) | 0.003 | 0.18 |  | 1.88(0.97-3.67) | 0.06 | 0.85 |  | 0.68(0.49-0.95) | 0.02 | 0.37 |
| Female | 0.11(<0.01-20.2) | 0.41 |  |  | 0.72(0.22-2.38) | 0.59 |  |  | 2.71(0.07-112.4) | 0.60 |  |  | 1.11(0.39-3.10) | 0.85 |  |
| Smoking status |  |  |  |  |  |  |  |  |  |  |  |  |  |  |  |
| Never | - | 0.96 | - |  | 0.84(0.02-32.3) | 0.93 | 0.73 |  | - | 0.92 | - |  | 1.83(0.16-21.4) | 0.63 | 0.43 |
| Ever | 0.56(0.29-1.10) | 0.09 |  |  | 1.61(1.15-2.25) | 0.005 |  |  | 1.81(0.96-3.42) | 0.07 |  |  | 0.68(0.49-0.93) | 0.02 |  |
| Stage |  |  |  |  |  |  |  |  |  |  |  |  |  |  |  |
| LD | 0.91(0.22-3.81) | 0.90 | 0.38 |  | 0.80(0.30-2.10) | 0.65 | 0.14 |  | 4.69(1.14-19.3) | 0.03 | 0.21 |  | 0.49(0.22-1.05) | 0.07 | 0.26 |
| ED | 0.44(0.21-0.92) | 0.03 |  |  | 1.75(1.21-2.53) | 0.003 |  |  | 1.69(0.82-3.48) | 0.16 |  |  | 0.79(0.55-1.12) | 0.19 |  |
| ECOG |  |  |  |  |  |  |  |  |  |  |  |  |  |  |  |
| 0-1 | 0.53(0.25-1.10) | 0.09 | 0.34 |  | 1.45(1.02-2.06) | 0.04 | 0.35 |  | 1.82(0.91-3.66) | 0.09 | 0.44 |  | 0.71(0.51-1.00) | 0.05 | 0.75 |
| 2 | 0.19(0.03-1.40) | 0.10 |  |  | 2.35(0.91-6.03) | 0.08 |  |  | 4.07(0.58-28.7) | 0.16 |  |  | 0.84(0.31-2.27) | 0.74 |  |
| Weight Loss |  |  |  |  |  |  |  |  |  |  |  |  |  |  |  |
| No | 0.36(0.16-0.80) | 0.01 | 0.29 |  | 1.28(0.86-1.90) | 0.23 | 0.10 |  | 1.52(0.73-3.17) | 0.27 | 0.21 |  | 0.83(0.58-1.20) | 0.33 | 0.48 |
| Yes | 0.81(0.22-2.94) | 0.75 |  |  | 2.48(1.26-4.88) | 0.01 |  |  | 4.07(1.05-15.8) | 0.04 |  |  | 0.63(0.32-1.22) | 0.17 |  |
| NSE |  |  |  |  |  |  |  |  |  |  |  |  |  |  |  |
| < 14.7 | 0.44(0.15-1.32) | 0.14 | 0.88 |  | 1.64(0.91-2.96) | 0.10 | 0.76 |  | 2.23(0.82-6.06) | 0.12 | 0.67 |  | 0.50(0.28-0.88) | 0.02 | 0.09 |
| ≥ 14.7 | 0.49(0.21-1.15) | 0.10 |  |  | 1.47(0.98-2.20) | 0.06 |  |  | 1.68(0.83-3.86) | 0.22 |  |  | 0.90(0.60-1.33) | 0.59 |  |
| Regimen |  |  |  |  |  |  |  |  |  |  |  |  |  |  |  |
| EP | 1.02(0.43-2.40) | 0.96 | 0.03 |  | 1.44(0.89-2.34) | 0.14 | 0.47 |  | 1.31(0.59-2.89) | 0.50 | 0.04 |  | 0.78(0.51-1.19) | 0.25 | 0.40 |
| IP | 0.23(0.08-0.65) | 0.01 |  |  | 1.85(1.14-3.00) | 0.01 |  |  | 5.38(1.74-16.6) | 0.004 |  |  | 0.60(0.39-0.93) | 0.02 |  |
| Second line Chemotherapy |  |  |  |  |  |  |  |  |  |  |  |  |  |  |  |
| No |  |  |  |  | 1.88(1.12-3.16) | 0.02 | 0.14 |  |  |  |  |  | 0.96(0.60-1.55) | 0.88 | 0.12 |
| Yes |  |  |  |  | 1.12(0.71-1.74) | 0.63 |  |  |  |  |  |  | 0.58(0.38-0.89) | 0.01 |  |
| Radiation to Tumor |  |  |  |  |  |  |  |  |  |  |  |  |  |  |  |
| No |  |  |  |  | 1.62(1.16-2.25) | 0.01 | 0.14 |  |  |  |  |  | 0.74(0.54-1.03) | 0.07 | 0.26 |
| Yes |  |  |  |  | 0.37(0.06-2.50) | 0.31 |  |  |  |  |  |  | 0.18(0.02-2.07) | 0.17 |  |
| Abbreviations: OR, odds ratios; CI, confidence intervals; HR, hazard ratio; *P_H_*, *P*-value for homogeneity test; LD, Limited disease; ED, Extensive disease; ECOG, Eastern Cooperative Oncology Group; NSE, neuron specific enolase; EP, etoposide-cisplatin ; IP, irinotecan-cisplatin  ^a^ OR, 95% CIs and corresponding *P*-values were calculated using multivariate Cox proportional hazard models, adjusted for the other variables.  ^b^ HR, 95% CIs and corresponding *P*-values were calculated using multivariate Cox proportional hazard models, adjusted for the other variables. | | | | | | | | | | | | | | | |

| Supplementary Table 4. In the extensive stage, combined effects of rs1058402G>A and rs763361C>T genotypes on chemotherapy response and survival outcomes. | | | | | | | | | | | | |
| --- | --- | --- | --- | --- | --- | --- | --- | --- | --- | --- | --- | --- |
|  |  | Chemotherapy response | | | |  | Overall survival | | |  | Progression-free survival | |
| No. of bad genotype^a^ | No. of case (%)^b^ | Responder  (%)^c^ | Non-responder  (%)^c^ | OR (95% CI)^d^ | *P*^d^ |  | MST (95% CI) | HR (95% CI)^e^ | *P*^e^ |  | HR (95% CI)^e^ | *P*^e^ |
|  |  |  |  |  |  |  |  |  |  |  |  |  |
| 0 | 86(45.7) | 69 (80.2) | 17 (19.8) | 1.00 |  |  | 11.2 (9.8-12.5) | 1.00 |  |  | 1.00 |  |
| 1 | 77(41.0) | 57 (74.0) | 20 (26.0) | 0.55 (0.28-1.06) | 0.07 |  | 8.0 (7.0-9.5) | 1.76 (1.19-2.58) | 0.004 |  | 1.52 (1.02-2.19) | 0.02 |
| 2 | 25(13.3) | 14 (56.0) | 11 (44.0) | 0.30 (0.12-0.75) | 0.01 |  | 7.0 (4.5-9.4) | 2.25 (1.33-3.82) | 0.003 |  | 1.49 (0.90-2.48) | 0.12 |
| *P*_trend_ |  |  |  | 0.55 (0.33-0.93) | 0.03 |  |  | 1.55 (1.21-1.98) | 6 × 10^-4^ |  | 1.28 (1.07-1.59) | 0.04 |
| Abbreviation: OR, odds ratio; CI, confidence interval; MST, median survival time (months); HR, hazard ratio.  ^a^bad genotype; rs1058402GA/AA and rs763361CC  ^b^Column percentage.  ^c^Row percentage.  ^d^OR, 95% CI, and their corresponding *P*-values were calculated by multivariate regression analysis, adjusted for age, gender, smoking status, stage, Eastern Cooperative Oncology Group performance status, weight loss, neuron specific enolase level, and first chemotherapy regimen.  ^e^HR, 95% CI and their corresponding *P*-values were calculated using multivariate Cox proportional hazard models, adjusted for age, gender, smoking status, stage, Eastern Cooperative Oncology Group performance status, weight loss, neuron specific enolase level, first chemotherapy regimen, second line chemotherapy, and radiation to primary tumor. | | | | | | | | | | | | |

| Supplement Table 5. Genotypes of *CD226* rs1790947 polymorphism and their association with the response to chemotherapy response and overall survival | | | | | | | | | | |
| --- | --- | --- | --- | --- | --- | --- | --- | --- | --- | --- |
|  |  | Chemotherapy response | | | |  | Overall survival | | | |
| Polymorphism | No. of case(%)^a^ | Responder  (%)^b^ | Non-responder  (%)^b^ | OR (95% CI)^c^ | *P*^c^ |  | MST (95% CI)^d^ | Log-Rank *P* | HR (95% CI)^d^ | *P*^d^ |
| rs1790947 |  |  |  |  |  |  |  |  |  |  |
| GG | 100 (38.9) | 65 (65.0) | 35 (35.0) | 1.00 |  |  | 8.44 (7.1-10.1) |  | 1.00 |  |
| GT | 121 (47.1) | 92 (76.0) | 29 (24.0) | 1.71 (0.93-3.14) | 0.08 |  | 9.81 (8.1-10.8) |  | 0.63 (0.46-0.87) | 0.01 |
| TT | 36 (14.0) | 30 (82.9) | 6( 17.1) | 2.88 (1.05-7.89) | 0.04 |  | 12.0 (8.8-15.2) | 0.01 | 0.60 (0.38-0.95) | 0.03 |
| Dominant |  |  |  | 1.91 (1.07-3.40) | 0.03 |  | 10.8 (9.5-12.0) | 0.01 | 0.62 (0.46-0.85) | 0.002 |
| Recessive |  |  |  | 2.20 (0.84-5.76) | 0.11 |  | 10.0 (8.8-10.8) | 0.06 | 0.79 (0.51-1.21) | 0.27 |
| Codominant |  |  |  | 1.70 (1.10-2.64) | 0.02 |  |  |  | 0.73 (0.58-0.92) | 0.01 |
| Abbreviation: OR, odds ratio; CI, confidence interval; MST, median survival time (months); HR, hazard ratio.  ^a^Column percentage.  ^b^Row percentage.  ^c^OR, 95% CI, and their corresponding *P*-values were calculated by multivariate regression analysis, adjusted for age, gender, smoking status, stage, Eastern Cooperative Oncology Group performance status, weight loss, neuron specific enolase level, and first chemotherapy regimen.  ^d^HR, 95% CI and their corresponding *P*-values were calculated using multivariate Cox proportional hazard models, adjusted for age, gender, smoking status, stage, Eastern Cooperative Oncology Group performance status, weight loss, neuron specific enolase level, first chemotherapy regimen second line chemotherapy and radiation to primary tumor. | | | | | | | | | | |
